# Impact of adherence to quality indicators and effects of targeted treatment with cefazolin or flucloxacillin on in-hospital mortality in patients with methicillin-susceptible Staphylococcus aureus (MSSA) bloodstream infections: a retrospective observational study

**DOI:** 10.1007/s15010-025-02473-4

**Published:** 2025-01-27

**Authors:** Sven Kalbitz, Kathrin Marx, Nils Kellner, Annette Glas, Maike Fedders, Christoph Lübbert

**Affiliations:** 1 Department of Infectious Diseases and Tropical Medicine, Hospital St. Georg, Leipzig, Germany; 2 Hospital Pharmacy, Hospital St. Georg, Leipzig, Germany; 3 Department of Laboratory Medicine, Hospital St. Georg, Leipzig, Germany; 4 Division of Infectious Diseases and Tropical Medicine, Department of Medicine I, Leipzig University Medical Center, Liebigstr. 20, D-04103 Leipzig, Germany; 5 Interdisciplinary Center for Infectious Diseases, Leipzig University Medical Center, Leipzig, Germany

**Keywords:** Methicillin-susceptible *Staphylococcus aureus* (MSSA), Bloodstream infection, Cefazolin, Flucloxacillin, Outcome, Quality indicators, Infectious disease (ID) consultation

## Abstract

**Purpose:**

To analyze the associations between adherence to quality indicators (QIs) in the treatment of bloodstream infections caused by methicillin-susceptible *Staphylococcus (S.) aureus* (MSSA) and in-hospital mortality.

**Methods:**

A retrospective observational study was conducted in patients admitted between 2019 and 2023 to Hospital St. Georg in Leipzig, Germany, with at least one positive blood culture for *S. aureus*. Ten QIs were categorized into four groups based on blood culture results, echocardiography, antibiotic treatment, and other parameters such as infectious disease (ID) specialist consultation. Propensity score (PS) matching was used to compare in-hospital mortality between MSSA patients treated with flucloxacillin and those treated with cefazolin. Multivariate Cox regression analysis was performed to determine risk factors associated with in-hospital mortality.

**Results:**

Of the 637 patients with *S. aureus* bloodstream infections, 495 patients with MSSA infection (77.8%) were included in the study. After the introduction of mandatory ID consultation in 2020, the median QI score increased to 9 out of a maximum of 10 points and was significantly higher in surviving cases than in fatal cases in subsequent years. There was a non-significant decrease in in-hospital mortality from 2019 (28.8%) to 2023 (22.7%) (*p* = 0.432). Based on PS matching, cefazolin had a favorable hazard ratio of 0.44 (95% CI 0.28–0.71; *p* < 0.001) for in-hospital mortality. The results of multivariate Cox regression analysis showed a significantly higher survival rate in patients who received QI-based management, including transesophageal echocardiography and antibiotic therapy initiated within 24 h.

**Conclusions:**

ID consultation is associated with better adherence to quality improvement measures. Targeted MSSA therapy with cefazolin, early initiation of antibiotic therapy, and adherence to antimicrobial treatment protocols increased survival rates in our study setting.

## Introduction

*Staphylococcus (S.) aureus* is the second most prevalent pathogen responsible for bloodstream infections (BSIs), followed by *Escherichia coli*. The incidence of *S. aureus* bloodstream infection (SABSI) is estimated to range from 9.3 to 65 cases per 100,000 inhabitants annually [1]. European Antimicrobial Resistance Surveillance Network (EARS-Net) data indicate a statistically significant increase in the incidence of *S. aureus* bloodstream infections (SABSIs) in the European Union (EU) and the European Economic Area (EEA) between 2005 and 2018 [2]. Data from Switzerland corroborate the observation of a 30% increase in the incidence of SABSIs between 2008 and 2021 [3]. The results of these studies indicate that the health burden of SABSIs is increasing in Europe. The mortality rate among patients with SABSIs is estimated to be between 19% and 40% [4].

A number of studies have demonstrated a correlation between adherence to quality indicators (QIs), including follow-up blood cultures, early intravenous (IV) administration of appropriate antimicrobial therapy and desired duration of therapy, and a reduction in 14- and 30-day mortality rates [5–7]. In a multicenter cohort study by Bai et al., the presence of an infectious disease (ID) consultation was associated with improved adherence to QIs, resulting in a reduction in in-hospital mortality and earlier discharge from the hospital for patients with SABSIs [8]. The IV administration of antibiotics with high anti-staphylococcal activity forms the basis of an appropriate treatment plan for patients diagnosed with methicillin-susceptible *S. aureus* (MSSA) bloodstream infection (BSI). The antistaphylococcal penicillins (ASPs) flucloxacillin, nafcillin and cloxacillin have been considered first-line agents for the treatment of MSSA BSI for decades [9]. Compared with ASPs, cefazolin, a first-generation cephalosporin, has been the subject of considerable debate in recent years [10–13]. There is currently no international consensus on the optimal treatment for MSSA BSI [14]. Meta-analyses have concluded that cefazolin is at least as effective as ASPs in the treatment of MSSA BSI and has a lower incidence of side effects [15, 16].

We investigated the management of MSSA BSIs before and after the introduction of mandatory ID consultation (2020) over a five-year period (2019 to 2023). The aim of the study was to analyze the relationship between adherence to QIs and in-hospital mortality, in particular regarding MSSA-specific antimicrobial treatment with flucloxacillin or cefazolin. Another key objective was to identify QIs relevant to in-hospital mortality.

## Patients and methods

### Setting

The Hospital St. Georg in Leipzig, Saxony, Germany comprises 1,066 beds and 25 different departments and clinics and is an academic teaching hospital of the University of Leipzig. Specialist departments for severe burns, septic surgery and infectious diseases/tropical medicine are important components of the tertiary care spectrum. Patients with infectious diseases are primarily cared for in two infectious disease wards, with a total of 44 beds, if intensive care therapy is not required.

### Study design and patients

This retrospective observational study included adult patients (aged 18 years or above) admitted between January 1, 2019 and December 31, 2023 who had at least one positive blood culture for *S. aureus*. Bacteremia was classified as community-acquired if the initial positive blood culture was obtained at the time of admission or within 72 h of admission, and as nosocomial if the initial positive blood culture was obtained after 72 h of admission. The estimated glomerular filtration rate (eGFR) was calculated using the Chronic Kidney Disease Epidemiology Collaboration (CKD-EPI) formula. The modified Charlson comorbidity index (mCCI) [17] was calculated to weight comorbidities. The case-mix index (CMI) was recorded as an indicator of the diversity, complexity and severity of patients treated in a healthcare facility. The SAPS II score was evaluated as a disease severity marker for ICU patients. Different biomarkers (leukocyte count, C-reactive protein [CRP], and procalcitonin [PCT]) were integrated into the analysis at the time of blood culture collection. All blood culture tests were conducted in the hospital microbiology department.

If treatment of SABSIs could not be carried out in one of the two infectious disease wards, an ID consultation was optional until 2019. In January 2020, a mandatory ID consultation was introduced for SABSIs. Concurrently, the attending ward physician was informed by the microbiologist by telephone. Within 24 hours, a comprehensive consultation was provided by a qualified ID physician. The term ‘complicated bacteremia’ was defined as the presence of infective endocarditis, septic metastases, implanted foreign material (e.g., prosthetic valves, cardiac devices, vascular grafts), a positive follow-up blood culture taken 48 to 96 h after the initiation of targeted antibiotic therapy, osteomyelitis, or a deep-seated abscess [18, 19]. Catheter-related infections were not classified as complicated if the catheter could be removed as a focus [20].

### Quality indicators (QIs)

Ten QIs for the management of SABSI, adapted from Fukushima et al. [7], were defined. The selected QIs are summarized in Table 1.

**Table 1:** Overview of the 10 QIs for the management of SABSIs, adapted from Fukushima et al. [7], and their point values

QI categories	Points
**A) Blood cultures**	
Follow-up blood cultures (after 48–72 h)	1
Consecutive confirmation of negative blood cultures	1
**B) Echocardiography (only one QI possible)**	
Transthoracic echocardiography (TTE)	1
Transesophageal echocardiography (TEE)	2
**C) Antibiotic treatment**	
Initial intravenous antibiotic therapy	1
Targeted antibiotic therapy (MSSA: cefazolin, flucloxacillin)	1
Initiation of antibiotic therapy within 24 h	1
Adherence to the defined duration of intravenous administration (14 days for uncomplicated and 28 days for complicated bacteremia)	1
**D) Other management**	
ID consultation	1
Description of SABSI in the medical discharge report	1

We assessed adherence to SABSI QIs for each included case by calculating points (ranging from 0 to10 points). Two points were awarded for performing transesophageal echocardiography (TEE) as the most sensitive and preferred method [21, 22], and one point was awarded for each of the other QIs. Some of the quality criteria described by Fukushima et al. [7] such as ‘dose adjustment to renal function’, ‘no intravenous-to-oral switch’, and ‘source control’ could not be assessed or could only be assessed incompletely due to the retrospective nature of the study. Therefore, the original criteria had to be adapted to the setting in our study.

### Antimicrobial therapy

Cefazolin (2 g IV every 6 to 8 h) or flucloxacillin (2 g IV every 4 to 6 h) were used as targeted therapies for MSSA BSIs according to internal guidelines. The cut-off time for the initiation of antimicrobial therapy was within 24 h of a positive blood culture result. In the case of bacterial coinfection, other antimicrobials were used based on their sensitivity, but these agents were not analyzed. An IV treatment period of 14 days was specified for uncomplicated bacteremia and 28 days for complicated bacteremia. Oral administration for complicated bacteremia as part of sequential therapy was carried out in close consultation with ID specialists. A specific evaluation of oral sequential therapy was not performed in this study.

### Exclusion criteria

SABSIs are associated with high mortality rates in patients hospitalized with COVID-19 [23, 24]. Importantly, the prevalence of *S. aureus* coinfections occurring after admission of patients for COVID-19 is likely related to a variety of patient- and environment-specific factors [24]. To avoid confounding effects, we excluded patients who also had polymerase chain reaction (PCR)-confirmed SARS-CoV-2 infection from the analysis.

Therefore, exclusion criteria were as follows: (1) patients aged < 18 years; (2) patients not hospitalized for at least 72 h; (3) patients who died before starting antibiotic administration; (4) patients discharged or transferred to other facilities within 72 h of the diagnosis of SABSI; (5) patients with SARS-CoV-2 infection; (6) patients with detection of methicillin-resistant *S. aureus* (MRSA); and (7) patients without targeted therapy in the context of palliative care. The 72-hour threshold was chosen because blood cultures for *S. aureus* were generally positive within 48 h, allowing sufficient time to select a targeted MSSA therapy.

### Ethics approval

The study was conducted in accordance with the ethical guidelines of the 1964 Declaration of Helsinki and its later amendments and was approved by the local ethics committee (Saxonian Board of Physicians, Dresden, Germany, vote EK-BR-37/21–1).

### Statistical analysis

Descriptive statistics were calculated for continuous variables, including means and standard deviations (SDs) or medians and interquartile ranges (IQRs), and for categorical variables, including numbers and percentages. These were used to characterize the patient cohort. Significance tests were performed using the chi-square test or Fisher’s exact test for categorical variables and t tests for continuous variables in cases where the data were normally distributed, or the Wilcoxon test otherwise. Q–Q plots were used to ensure normality of the data. A p-value of less than or equal to 0.05 was considered statistically significant.

Propensity score (PS) matching was used to represent the characteristics of the cefazolin- and flucloxacillin-treated patient groups. Propensity scores were estimated from available data using a logistic regression model in which MSSA BSI therapy was the dependent variable and patient characteristics at baseline were the independent covariates. These included age, sex, type of acquisition (community-acquired/nosocomial), type of ward, severity of infection (complicated/uncomplicated bacteremia), site of infection, comorbidities, implanted foreign material, CMI and biomarkers (leukocyte count, CRP, and PCT). For PS matching, each patient treated with flucloxacillin was matched to a patient treated with cefazolin (1:1). To minimize confounding effects, an exact match was defined for the number of patients in the ICU.

The standardized mean difference (SMD) was used to represent the differences between groups. A regression model was constructed to investigate the impact of targeted MSSA therapy (cefazolin, flucloxacillin) on in-hospital survival rates. The Kaplan‒Meier method was used to calculate survival curves and to compare significance using the log-rank test. Hazard ratios (HRs) with 95% confidence intervals (95% CI) were used as common measures to assess relative risk. Multivariate Cox regression analysis was performed to evaluate the influence of multiple factors on in-hospital mortality, considering all parameters that were significant in univariate analyses (*p* < 0.05). Statistical analyses were performed using R statistical software (version 4.4.1).

## Results

### Patients

**Fig. 1:**
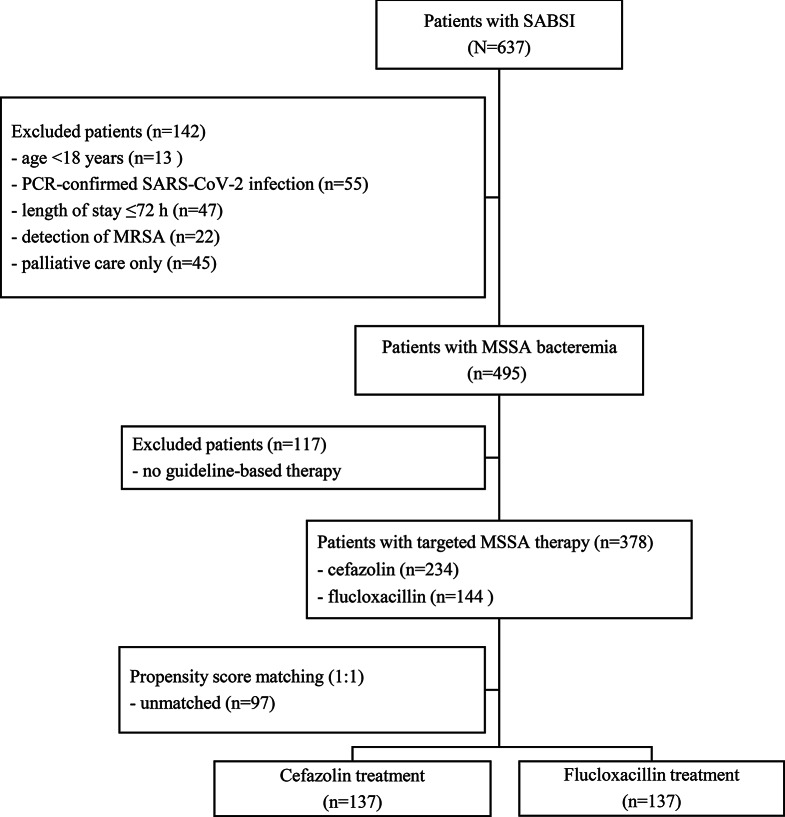
Flowchart of the study population

Over the course of the observation period, a total of 637 patients were hospitalized with SABSIs. A summary of the demographic and clinical characteristics is provided in **Supplementary Table S1**. In total, 615 MSSA cases (96.5%) and 22 MRSA cases (3.5%) were identified. The incidence rates of MSSA BSI (defined as MSSA BSI per 1,000 inpatient cases per year) notably changed over the course of the study period, with rates observed as follows: 3.2 in 2019, 3.3 in 2020, 3.7 in 2021, 4.5 in 2022, and 3.5 in 2023. Fifty-five patients had PCR-confirmed SARS-CoV-2 infection and were therefore excluded from further analyses. The application of other exclusion criteria is shown in Fig. 1; multiple exclusion criteria may apply to a single case.

The baseline characteristics of the remaining 495 patients with MSSA bacteremia from 2019 to 2023 are presented in Table 2 and were stratified by year. The mean age was 70 years, and 71.5% (354/495) of the patients were male. The time trend analysis revealed significant changes in several variables, including sex (*p* = 0.030), Charlson comorbidity index (*p* = 0.005), site of infection (*p* = 0.023), implanted foreign material (*p* = 0.003), and certain comorbidities such as cancer (*p* = 0.004) and liver disease (*p* < 0.001). The most common site of infection overall was the skin and soft tissue (221/495, 44.6%).

**Table 2:** Baseline characteristics of patients with MSSA bacteremia (*n* = 495) at Hospital St. Georg from 2019 to 2023, stratified by year

Year	2019	2020	2021	2022	2023	Total
Number of patients (n)	119	101	86	101	88	495
Age (years)	72.4 (14.0)	69.7 (15.2)	70.3 (14.2)	68.3 (17.5)	71.3 (15.7)	70.4 (15.4)
Age ≥ 65 years	88 (73.9)	71 (70.3)	55 (64.0)	66 (65.3)	60 (68.2)	340 (68.7)
Male sex	75 (63.0)	70 (69.3)	69 (80.2)	70 (69.3)	70 (79.5)	354 (71.5)
mCCI	3.0 (1.0, 4.0)	3.0 (1.0, 6.0)	4.0 (2.0, 6.0)	3.0 (1.0, 4.0)	3.5 (1.7, 5.2)	3.0 (1.0, 5.0)
Length of hospital stay (days)	27.5 (22.5)	33.3 (28.2)	26.4 (25.9)	27.8 (20.7)	24.2 (21.3)	28.0 (23.9)
Intensive care unit (ICU)	42 (35.3)	40 (39.6)	31 (36.0)	33 (32.7)	23 (26.1)	169 (34.1)
eGFR CKD-EPI (mL/min/1.73 m^2^)	59.8 (33.2)	59.5 (33.6)	55.1 (34.9)	57.5 (34.8)	52.6 (33.4)	57.2 (33.9)
Complicated bacteremia	39 (32.8)	41 (40.6)	21 (24.4)	34 (33.7)	25 (28.4)	160 (32.3)
Type of acquisition						
Community-acquired	43 (36.1)	33 (32.7)	39 (45.3)	43 (42.6)	42 (47.7)	200 (40.4)
Nosocomial infection	76 (63.9)	68 (67.3)	47 (54.7)	58 (57.4)	46 (52.3)	295 (59.6)
Site of infection						
Endocarditis	9 (7.6)	8 (7.9)	1 (1.2)	10 (9.9)	5 (5.7)	33 (6.7)
Skin and soft tissue	47 (39.5)	39 (38.6)	37 (43.0)	51 (50.5)	47 (53.4)	221 (44.6)
Catheter-related	10 (8.4)	5 (5.0)	12 (14.0)	4 (4.0)	12 (13.6)	43 (8.7)
Bone and joint	10 (8.4)	10 (9.9)	6 (7.0)	9 (8.9)	1 (1.1)	36 (7.3)
Meningitis	0 (0.0)	0 (0.0)	0 (0.0)	1 (1.0)	0 (0.0)	1 (0.2)
Respiratory tract	2 (1.7)	0 (0.0)	1 (1.2)	1 (1.0)	0 (0.0)	4 (0.8)
Port infection	10 (8.4)	6 (5.9)	11 (12.8)	3 (3.0)	1 (1.1)	31 (6.3)
Spondylodiscitis/ osteomyelitis	8 (6.7)	10 (9.9)	10 (11.6)	4 (4.0)	6 (6.8)	38 (7.7)
Unknown foci	23 (19.3)	23 (22.8)	8 (9.3)	18 (17.8)	16 (18.2)	88 (17.8)
Comorbidity						
Cancer	13 (10.9)	20 (19.8)	23 (26.7)	10 (9.9)	10 (11.4)	76 (15.4)
Chronic kidney disease	31 (26.1)	20 (19.8)	22 (25.6)	22 (21.8)	33 (37.5)	128 (25.9)
Diabetes	57 (47.9)	42 (41.6)	33 (38.4)	43 (42.6)	41 (46.6)	216 (43.6)
Liver disease	4 (3.4)	19 (18.8)	23 (26.7)	18 (17.8)	19 (21.6)	83 (16.8)
Implanted foreign material	61 (51.3)	32 (31.7)	31 (36.0)	27 (26.7)	32 (36.4)	183 (37.0)

**Abbreviations**: eGFR, estimated glomerular filtration rate (GFR, mL/min/1.73 m^2^); mCCI, modified Charlson comorbidity index. Data are presented as numbers (percentages), mean (SD) or median values (IQR: Q1-Q3)

### Relationship between in-hospital mortality and QI adherence in the treatment of MSSA BSIs

**Fig. 2:**
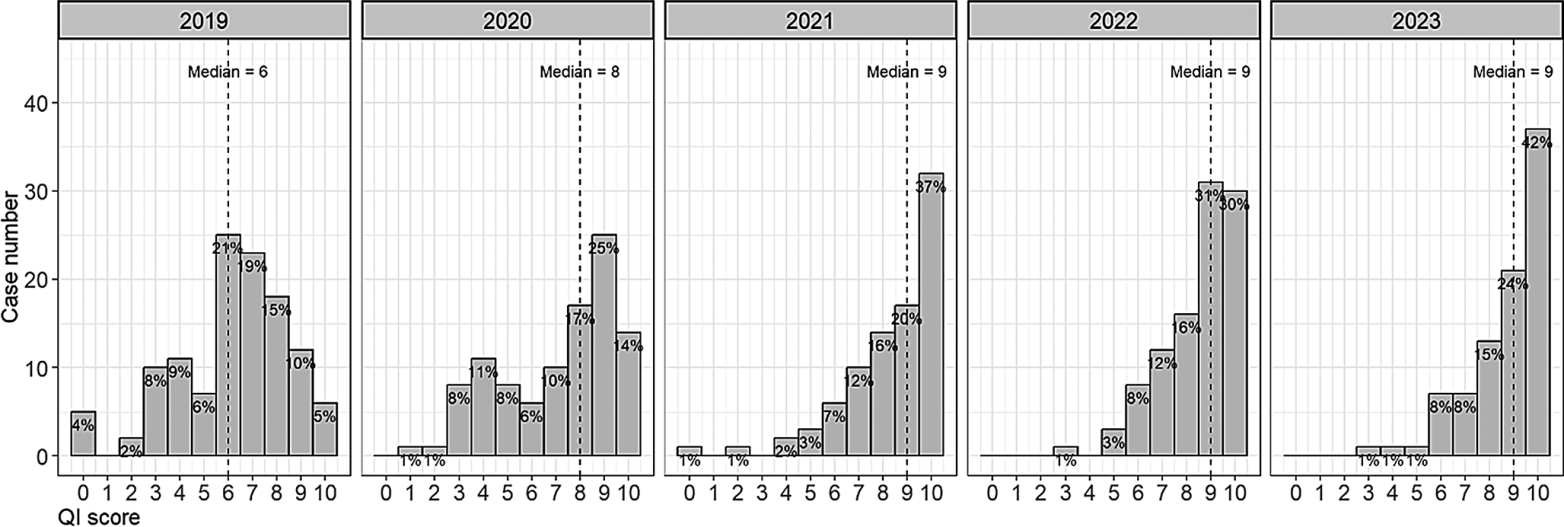
Distribution of QIs for all MSSA BSI cases, stratified by year (*n* = 495)

**Fig. 3:**
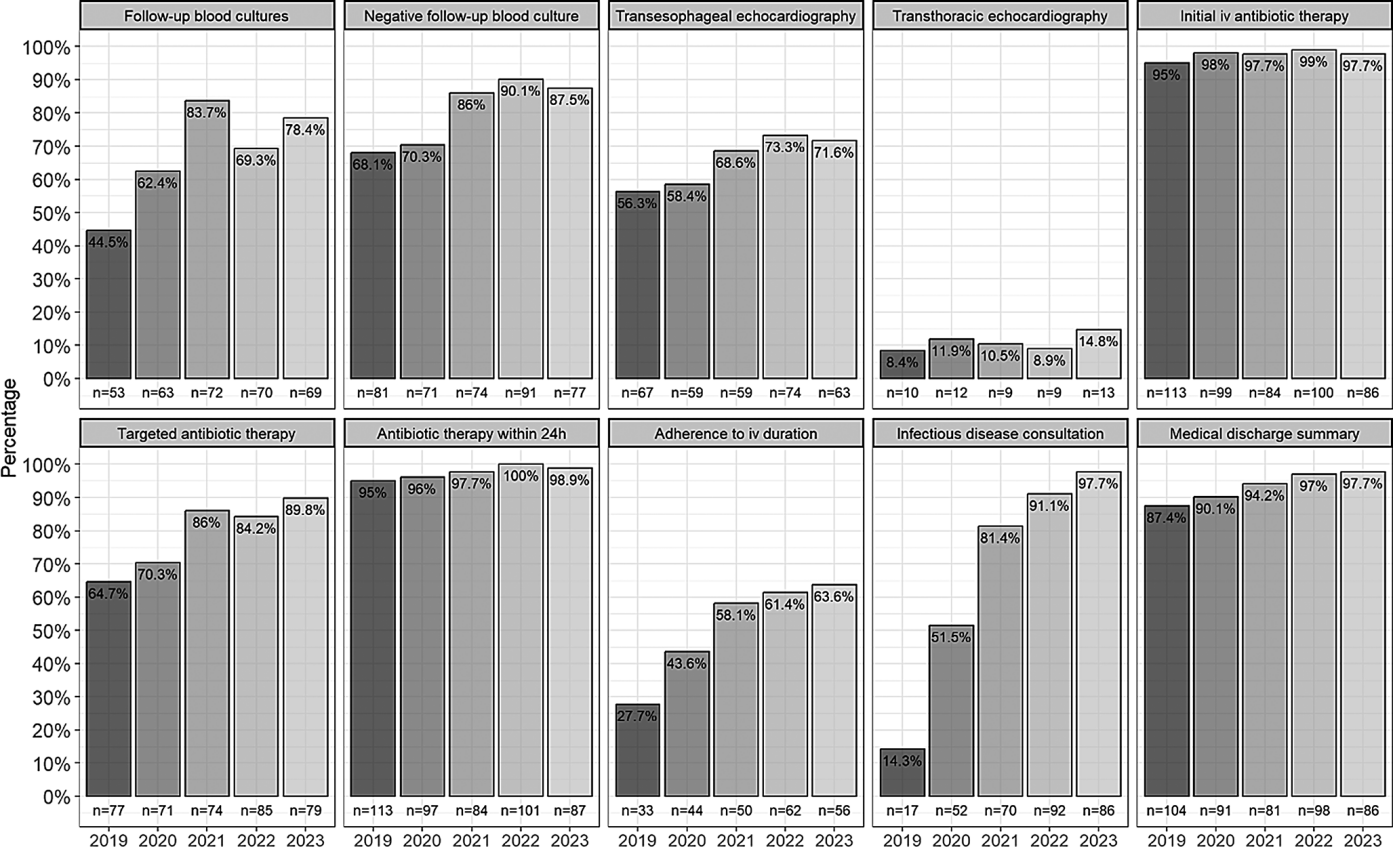
Adherence to QIs, stratified by year (*n* = 495)

Following the introduction of mandatory ID consultation in 2020, the median QI score increased to a maximum of 9 points in subsequent years (Fig. 2). The adherence rates for each QI are shown in Fig. 3 and are stratified by year. The implementation of almost all QIs increased over the 5-year period studied. In particular, between 2019 and 2023, there were significant developments, i.e. before and after mandatory ID consultation, in the following QI categories: follow-up blood cultures (44.5–78.4%; *p* < 0.001), consecutive confirmation of negative blood cultures (68.1–87.5%, *p* = 0.002), TEE (56.3–71.6%, *p* = 0.035), targeted antibiotic therapy (64.7–89.8%, *p* < 0.001), adherence to recommended IV duration (27.7–63.6%, *p* < 0.001), ID specialist consultation (14.3–97.7%, *p* < 0.001), and medical discharge summary (87.4–97.7%, *p* = 0.015).

### QI scores and patient outcomes

**Fig. 4:**
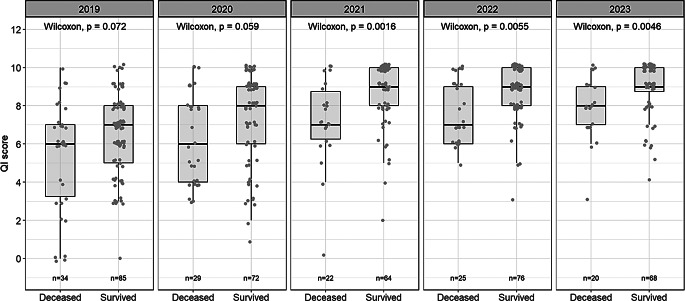
Comparison of QI scores between survived and deceased MSSA BSI cases (*n* = 495)

**Fig. 5:**
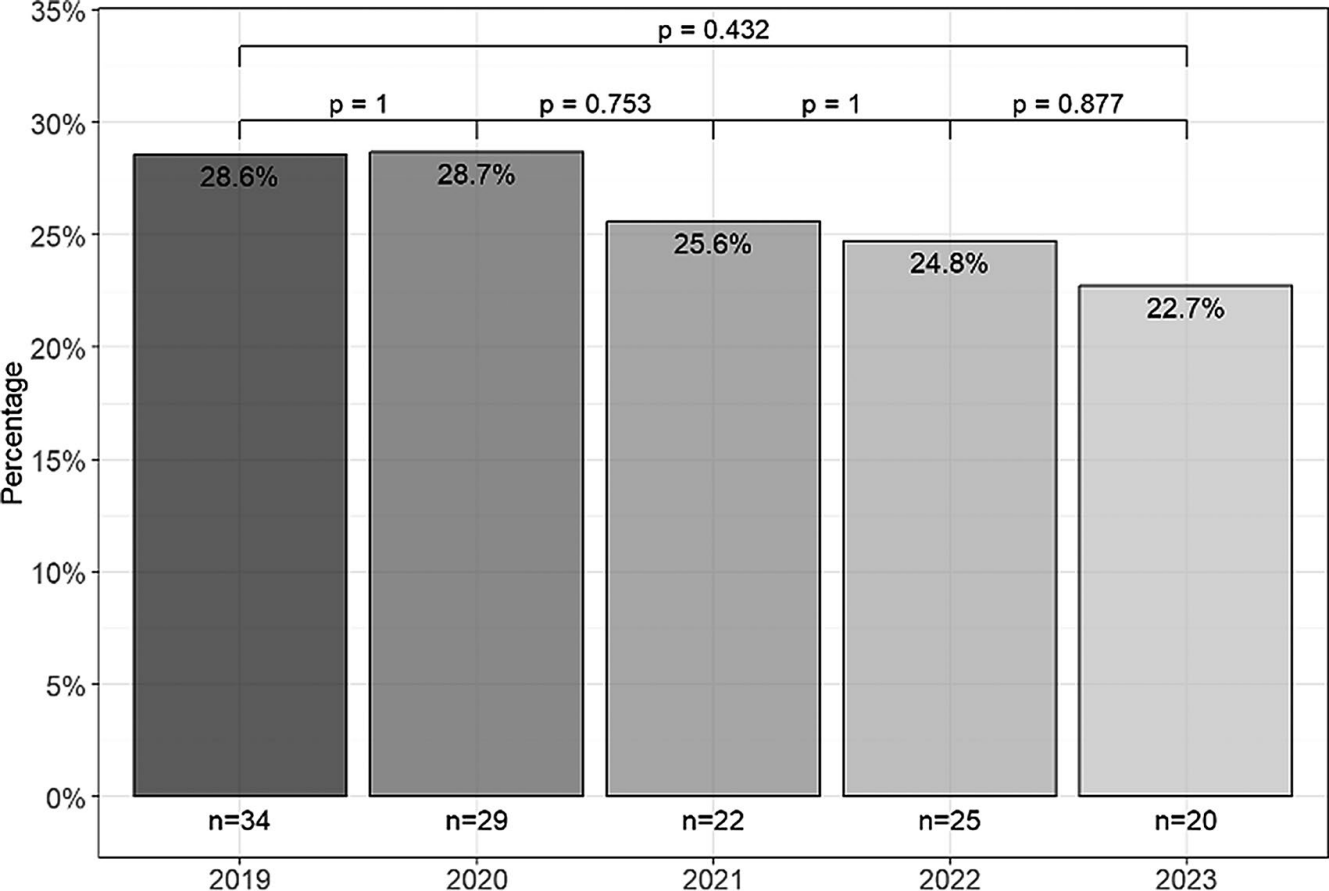
Comparison of the in-hospital mortality stratified by years

QI scores were compared between deceased and surviving patients across all MSSA BSI cases, stratified by year (Fig. 4). The median QI scores [interquartile ranges, IQRs: Q1-Q3] of deceased vs. surviving patients were 6 [3.8] vs. 7 [3.0] in 2019, 6 [4.0] vs. 8 [3.0] in 2020, 7 [2.5] vs. 9 [2.0] in 2021, 7 [3.0] vs. 9 [2.0] in 2022, and 8 [2.0] vs. 9 [1.2] in 2023. As of 2021, QI scores in the group of deceased patients were significantly lower than in the group of survivors (*p* < 0.05). A comparison of in-hospital mortality rates stratified by year is shown in Fig. 5. There was a non-significant decrease (*p* = 0.432) in in-hospital mortality from 2019 (28.8%) to 2023 (22.7%).

The overall adherence to QIs and the patient outcomes are shown in Table 3. To avoid immortal time bias, we established landmark times for each time-dependent QI. The analyses were performed only for patients who were alive at day 3 (for follow-up blood cultures within 72 h), day 10 (for IV treatment duration in patients with uncomplicated MSSA BSI), and day 28 (for IV treatment duration in patients with complicated MSSA BSI) [36]. In univariate analysis, the adherence to five QIs, including (1) consecutive confirmation of negative blood cultures, (2) TEE, (3) initial IV antibiotic therapy, (4) start of antibiotic therapy within 24 h, and (5) ID specialist consultation, was associated with a significantly lower hazard ratio (HR) for in-hospital mortality.

**Table 3:** Clinical outcomes of patients with MSSA BSIs (*n* = 495), stratified by QIs

	Number of patients		Number of deaths		HR (95% CI)
Adherence to QIs	Yes	No	Yes	No	
Follow-up blood cultures within 72 hours^a^	323 (67.2)	158 (32.8)	82 (25.4)	34 (21.5)	1.06 (0.71–1.59)
Consecutive confirmation of negative blood cultures	394 (79.6)	101 (20.4)	87 (22.1)	43 (42.6)	0.47 (0.32–0.67)
Transthoracic echocardiography (TTE)	53 (10.7)	442 (89.3)	15 (28.3)	115 (26.0)	1.06 (0.62–1.82)
Transesophageal echocardiography (TEE)	322 (65.1)	173 (34.9)	61 (18.9)	69 (39.9)	0.33 (0.23–0.47)
Initial intravenous antibiotic therapy	482 (97.4)	13 (2.6)	122 (25.3)	8 (61.5)	0.16 (0.07–0.32)
Targeted antibiotic therapy (MSSA: cefazolin, flucloxacillin)	378 (76.4)	117 (23.6)	96 (25.4)	34 (29.1)	0.8 (0.54–1.2)
Start of antibiotic therapy within 24 h	482 (97.4)	13 (2.6)	123 (25.5)	7 (53.8)	0.26 (0.12–0.56)
Adherence to the recommended duration of IV administration^b^	230 (56.8)	175 (43.2)	22 (9.5)	18 (10.3)	0.83 (0.46–1.93)
ID consultation	317 (64.0)	178 (36.0)	73 (23.0)	57 (32.0)	0.65 (0.46–0.93)
Description of SABSI in the medical discharge report	460 (92.9)	35 (7.1)	117 (25.4)	13 (37.1)	0.86 (0.48–1.54)

***Abbreviations*** HR, hazard ratio; CI, confidence interval; QI, quality indicator. Data are presented as numbers (percentages)

^a^ Patients who survived at least 3 days were included (*n* = 481)

^b^ Patients who survived at least 10 days (uncomplicated bacteremia) or 28 days (complicated bacteremia) were included (*n* = 405)

### Dependence of in-hospital mortality on targeted MSSA therapy with cefazolin or flucloxacillin

**Fig. 6:**
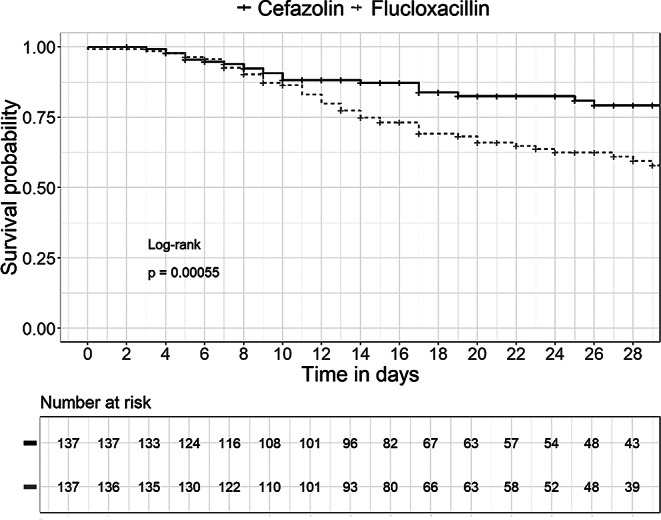
Kaplan‒Meier survival curves, differentiated by targeted therapy of MSSA bacteremia (cefazolin vs. flucloxacillin) over an observation period of 28 treatment days

Supplementary Table S2 shows the baseline characteristics of the cefazolin group compared to the flucloxacillin group in *n* = 378 patients with targeted MSSA therapy. Cefazolin was the most commonly used MSSA antimicrobial with 61.9% of administrations (234/378). Flucloxacillin (*n* = 144) was preferred in patients with complicated infections (flucloxacillin vs. cefazolin, 45% vs. 33%), in patients with implanted foreign material (flucloxacillin vs. cefazolin, 45% vs. 33%), and in intensive care patients (flucloxacillin vs. cefazolin, 45% vs. 25%). The mean duration of hospitalization and IV treatment for MSSA did not differ significantly between both groups. By propensity score matching (1:1; with exact matching of patients in the intensive care unit), 137 cefazolin cases were matched with an equal number of flucloxacillin cases. Detailed clinical characteristics of the matched patients are shown in Supplementary Table S2. Twenty-four covariates met a threshold of mean differences < 0.1. Four covariates (age, length of hospital stay, CRP and PCT) could not be balanced. CRP was the variable with the largest mean difference (0.223). Figure 6 shows the results of the Kaplan-Meier survival analyses for targeted therapy of MSSA.

The survival analysis revealed significantly lower in-hospital mortality for patients receiving cefazolin therapy (HR 0.44, 95% CI 0.28–0.71; *p* < 0.001). The in-hospital mortality rate was 39.4% (54/137) in the flucloxacillin group and 19.7% (27/137) in the cefazolin group. This effect was confirmed in all subgroup analyses; the results are summarized in **Supplementary Table S3**. In addition, a multivariate Cox regression analysis in the full cohort (*n* = 495) was performed to adjust for all significant parameters from the univariate analyses (*p* < 0.05) that were associated with in-hospital mortality in MSSA BSI patients (Table 4). Performing TEE and initiation of antibiotic therapy within 24 hours were associated with lower in-hospital mortality, whereas antimicrobial therapy with flucloxacillin was not. Table 4 summarizes the statistically significant parameters influencing in-hospital mortality in the multivariate analysis.

**Table 4:** Statistically significant results of the multivariate Cox regression analysis of in-hospital mortality (*n* = 495)

Parameter	HR (95% CI)	*P* value
**Patients characteristics**		
Age ≥ 65 years	2.14 (1.32–3.47)	0.002
mCCI ≥ 3 points	1.73 (1.10–2.74)	0.018
**Site of infection**		
Port	2.11 (1.03–4.30)	0.040
Endocarditis	2.50 (1.30–4.82)	0.006
Unknown foci	2.18 (1.28–3.73)	0.004
**MSSA-treatment**		
Flucloxacillin	2.04 (1.40–2.98)	< 0.001
**Comorbidity**		
Chronic kidney disease	1.50 (1.00-2.28)	0.050
**SAB QI categories**		
Transesophageal echocardiography (TEE)	0.38 (0.22–0.50)	< 0.001
Start of antibiotic therapy within 24 h	0.31 (0.14–0.69)	0.008

*Abbreviations*: mCCI, modified Charlson comorbidity index; HR, hazard ratio; CI, confidence interval; QI, quality indicator

## Discussion

This large retrospective study from a single tertiary center including *n* = 495 patients with MSSA bacteremia shows that adherence to QIs is a fundamental aspect of the management of SABSI. As expected, the introduction of mandatory ID consultation for confirmed MSSA BSI during 2020 led to a significant increase in ID specialist consultations from 14.3% in 2019 to 97.7% in 2023 (*p* < 0.001) in our study. The median QI score increased to 9 out of a maximum of 10 points. In addition, time trend analyses showed significant developments from 2019 to 2023 in 7 out of 10 QIs. Consultation with ID specialists has been documented in numerous studies as a core element of the management of patients with SABSI [8, 25–27]. In our study, increasing the number of QI categories achieved was associated with a non-significant reduction in the mortality rate among the *n* = 495 MSSA BSI cases from 28.8% in 2019 to 22.7% in 2023. A significant difference was found in QI scores between the group of deceased patients and the group of survivors in the period since 2021 (median: 7 vs. 9, *p* < 0.05). The study by Fukushima et al. [7], proposing QIs that served as a basis for our own study, also reported that lower QI scores were associated with a poor prognosis and that QIs were significantly lower in deceased patients (median: 7 vs. 9, *p* < 0.01). These data demonstrate the clinical importance of adherence to QIs in terms of improving patient outcomes [6–8, 28, 29]. As the reduction in in-hospital mortality observed in our study was not statistically significant, we also investigated the effects of implementing individual QI categories on in-hospital mortality. For this purpose, we performed a univariate Cox regression analysis with all MSSA BSI cases. This analysis revealed that the implementation of 5 of the 10 QI categories was significantly associated with a reduction of in-hospital mortality, including (1) consecutive confirmation of negative blood cultures, (2) TEE, (3) initial intravenous antibiotic therapy, (4) initiation of antibiotic therapy within 24 h, and (5) consultation with an ID specialist. In particular, analyzing the QI for adherence to the defined duration of IV administration was challenging. To avoid immortal time bias, we excluded patients who died before a certain time point (landmark analysis).

However, the optimal duration of IV therapy is still controversial [19, 30, 31]. Two randomized controlled trials are currently underway to evaluate the optimal duration of antimicrobial treatment: the SAB-7 trial, which aims to determine whether 7 days of antibiotic treatment is non-inferior to 14 days of antibiotic treatment in patients with uncomplicated SABSI [32], and the SAFE trial, which compares 4 and 6 weeks of IV antibiotic therapy in patients with complicated SABSI [33].

In our study, TEE was associated with a lower risk of death (HR 0.33, 95% CI 0.23–0.47; *p* < 0.001). However, it is not possible to determine with certainty whether the performance of TEE alone is directly associated with a lower risk of mortality [34, 35], since the improvement in clinical outcomes due to improved adherence to QI categories is most likely multifactorial. This makes it difficult to determine the impact of individual QI changes on treatment outcomes in SABSI patients, as the interaction between different management elements makes this assessment complex [8]. As already shown by Escrihuela-Vidal et al., it is not the individual QI but the bundle of quality indicators that improves survival [36]. This is because individual indicators may imply compliance with other factors.

Another important finding of our study was the highly significant difference in in-hospital mortality between cefazolin (19.7%) and flucloxacillin (39.4%) in the treatment of MSSA bacteremia (*p* < 0.001). To account for the possible preference for flucloxacillin over cefazolin in severe infections with high inoculum and other confounding factors, we performed a propensity score analysis (1:1). Since flucloxacillin was preferred in patients requiring intensive care, we performed an exact matching of ICU patients to minimize selection bias. However, a discernible difference remained, as patients receiving flucloxacillin suffered most likely from more severe disease, as reflected by a higher SAPS II score, a higher endocarditis rate and a higher mean PCT. The survival analysis revealed a significant benefit of cefazolin therapy with a HR of 0.44 (95% CI 0.28–0.71, *p* < 0.001). To identify factors associated with in-hospital mortality, we also performed a multivariate Cox regression analysis with all MSSA patients (*n* = 495). There was a significantly greater risk for in-hospital mortality in patients treated with flucloxacillin (HR 2.04, 95% CI 1.40–2.98; *p* < 0.001), patients aged ≥ 65 years (HR 2.14, 95% CI 1.32–3.47; *p* = 0.002), patients with a mCCI ≥ 3 points (HR 1.73, 95% CI 1.10–2.74; *p* = 0.018), patients with chronic kidney disease (HR 1.50, 95% CI 1.00–2.28; *p* = 0.05), and patients with infective endocarditis, infection of an endovascular port system or unknown focus of infection. Thus, our study confirms the relevance of mortality factors mentioned in the literature, particularly older age, comorbidities and site of infection [37–40]. Furthermore, our study results demonstrate a strong clinical advantage of cefazolin over ASPs in the treatment of MSSA bacteremia, which is consistent with results of other studies comparing cefazolin with ASPs [12, 41, 42]. A meta-analysis by Bidell et al. based on data from seven studies found a significant reduction in mortality associated with cefazolin therapy (OR 0.63, 95% CI 0.41–0.99; *p* = 0.05) in patients with MSSA bacteremia compared with ASPs [43]. Another meta-analysis of 14 non-randomized trials by Rao et al. which estimated relative risks (RRs) also found a lower 30-day mortality with cefazolin treatment (RR 0.70; 95% CI 0.54–0.91, *p* = 0.12) [44]. Furthermore, cefazolin was not associated with increased treatment failure rates in the treatment of deep-seated MSSA BSI and was considered a viable alternative to oxacillin. A large meta-analysis of retrospective studies published in 2019 showed that cefazolin is at least as effective as ASP with a lower incidence of nephrotoxicity [15]. In conclusion, cefazolin was therefore recommended as first-line therapy for MSSA bacteremia in a recent clinical review by Lam and Stokes [45].

### Limitations

In light of the absence of randomization and control in the study design, there was a possibility of unanticipated confounding factors. Importantly, retrospective observational studies are particularly susceptible to selection bias, which cannot be fully eliminated by multivariate analyses and adjustments using propensity scores. Furthermore, a time bias cannot be ruled out, especially for time-dependents QIs (follow-up blood cultures after 48–72 h and the adherence to the defined duration of IV administration). To control for these, we excluded patients who died before a specific landmark time. As the study was retrospective in nature, only in-hospital mortality could be evaluated. In evaluating the constraints of this study, it is essential to acknowledge that the medical records did not incorporate source control as a quality indicator for the management of SABSIs. In addition, the cases examined had a considerable drop-out rate of 23.6% (neither flucloxacillin nor cefazolin were administered). However, in a retrospective observational Canadian study by Bai et al., an even higher drop-out rate of 49% was reported [42]. Moreover, the ID consultation was only advisory in nature; the ID specialist consulted was not authorized to give binding instructions for patients who were not treated in ID wards.

## Conclusions

The findings of our study indicate that higher adherence to QIs is associated with superior patient outcomes. In particular, targeted therapy with cefazolin and early initiation of antimicrobial therapy were identified as key factors that significantly increased the survival rate within the hospital setting. The results of this retrospective study suggest the need for further investigation through randomized controlled trials to corroborate the aforementioned clinical benefit.

## Data Availability

No datasets were generated or analysed during the current study.
